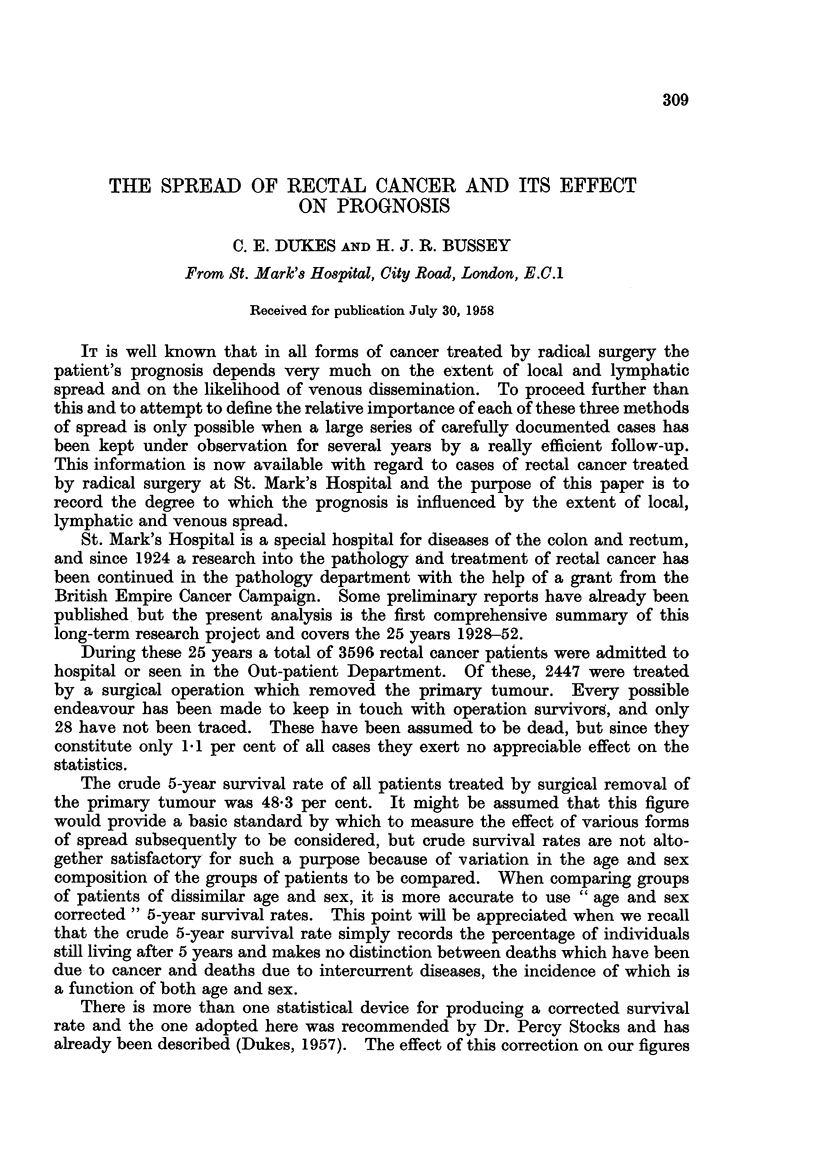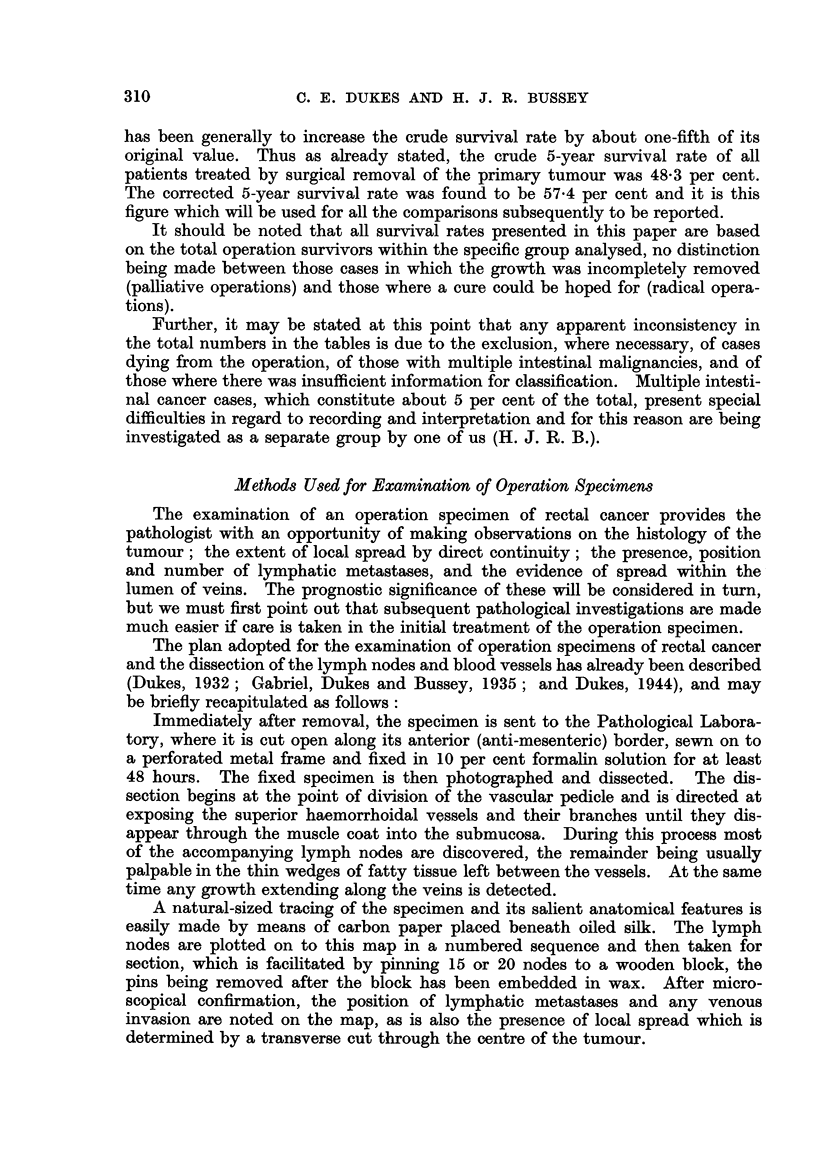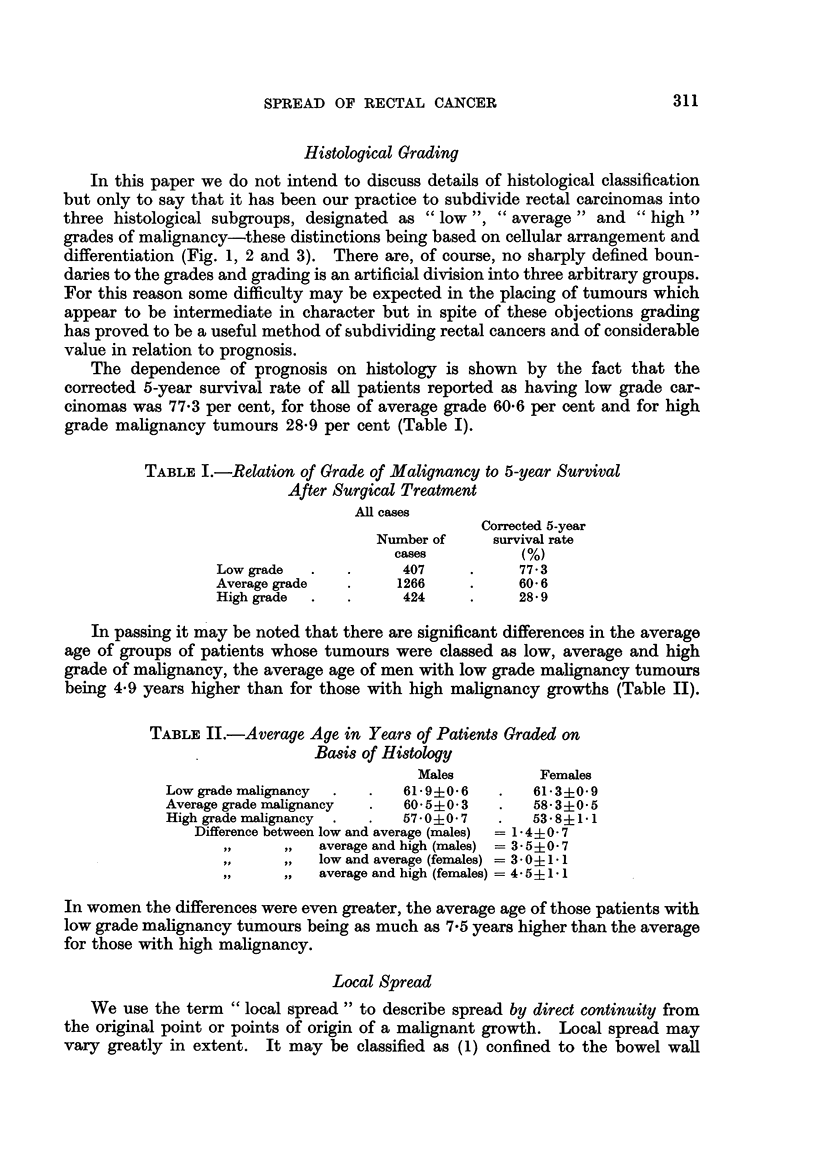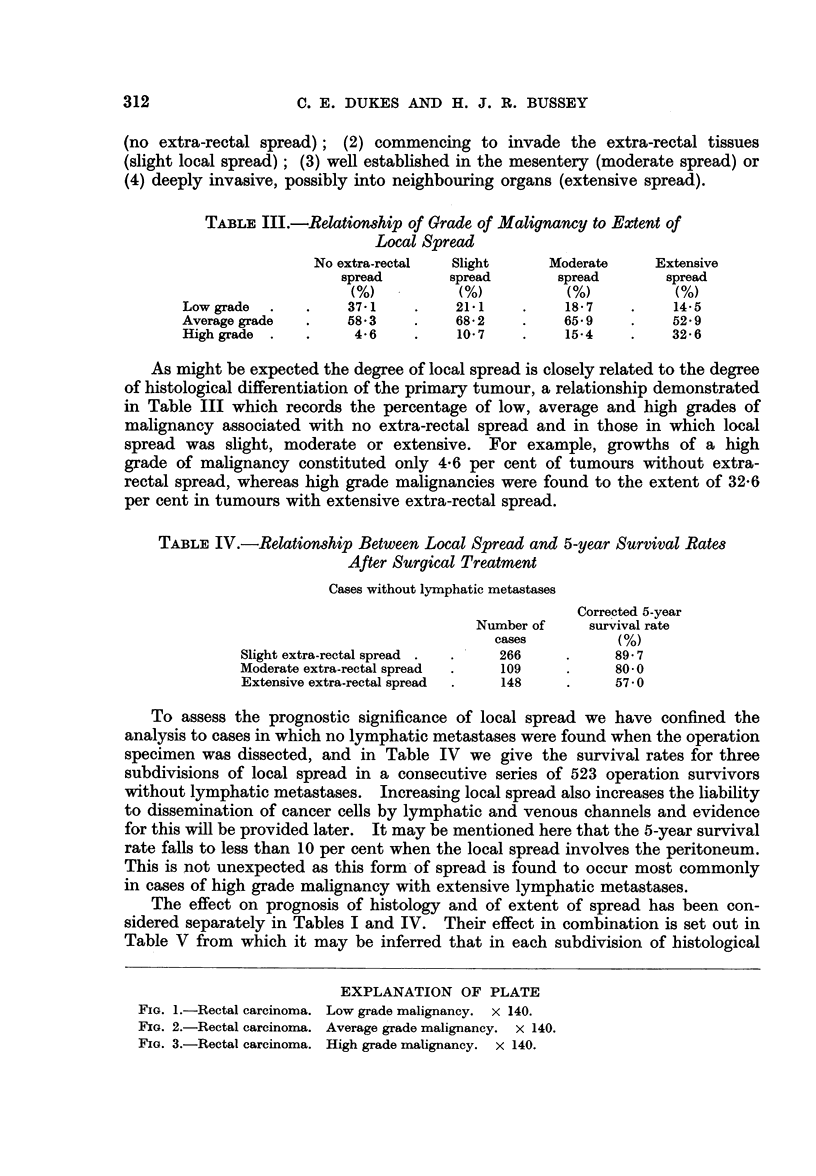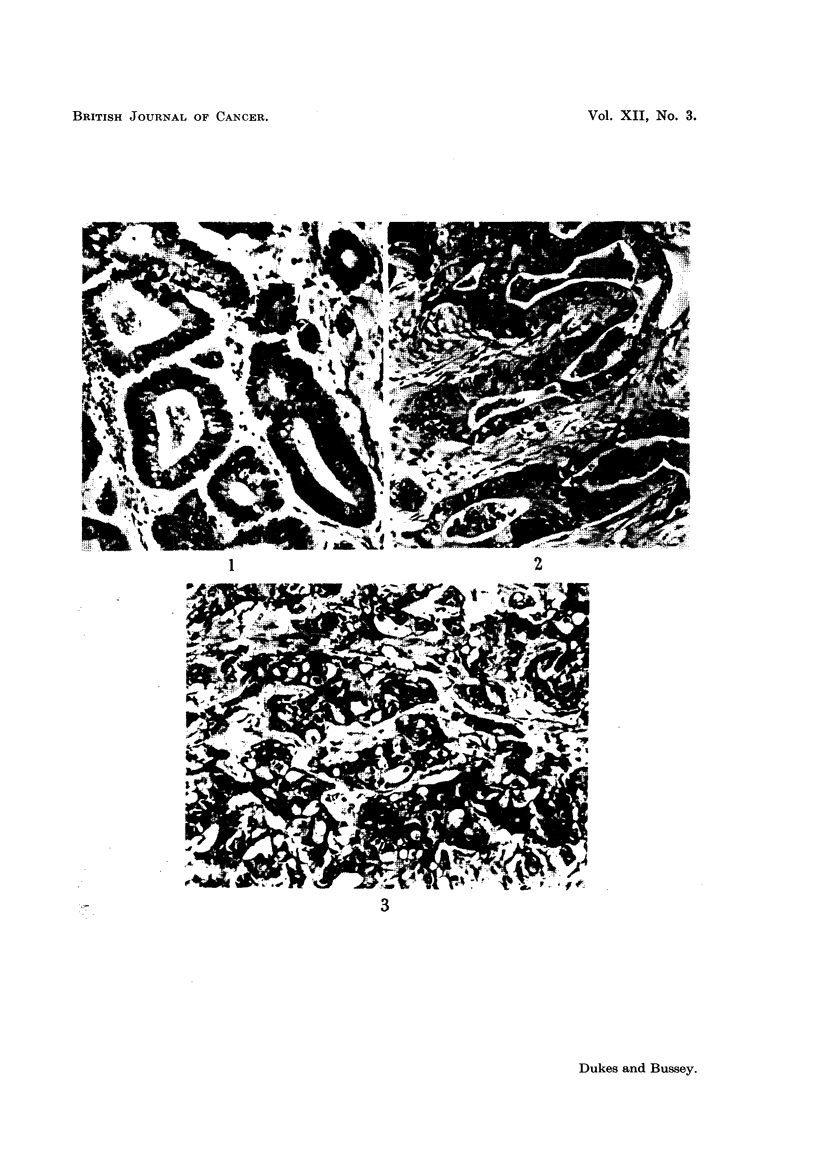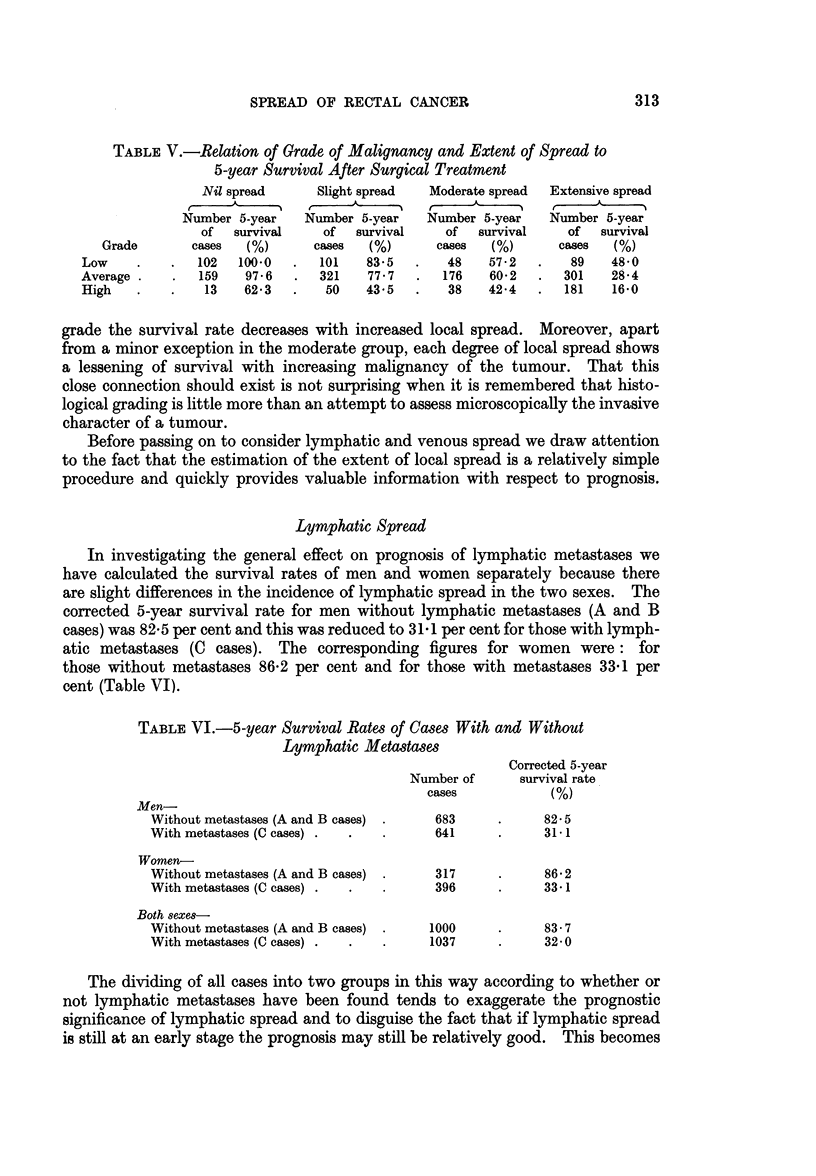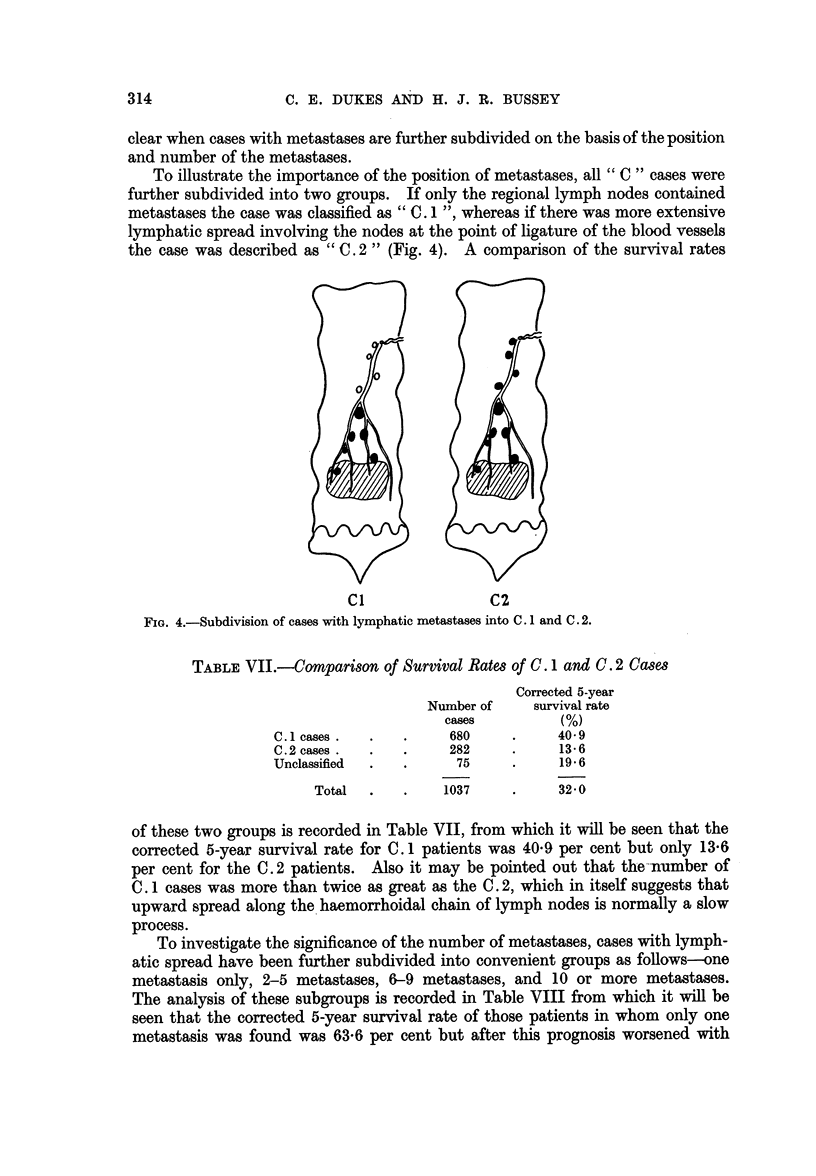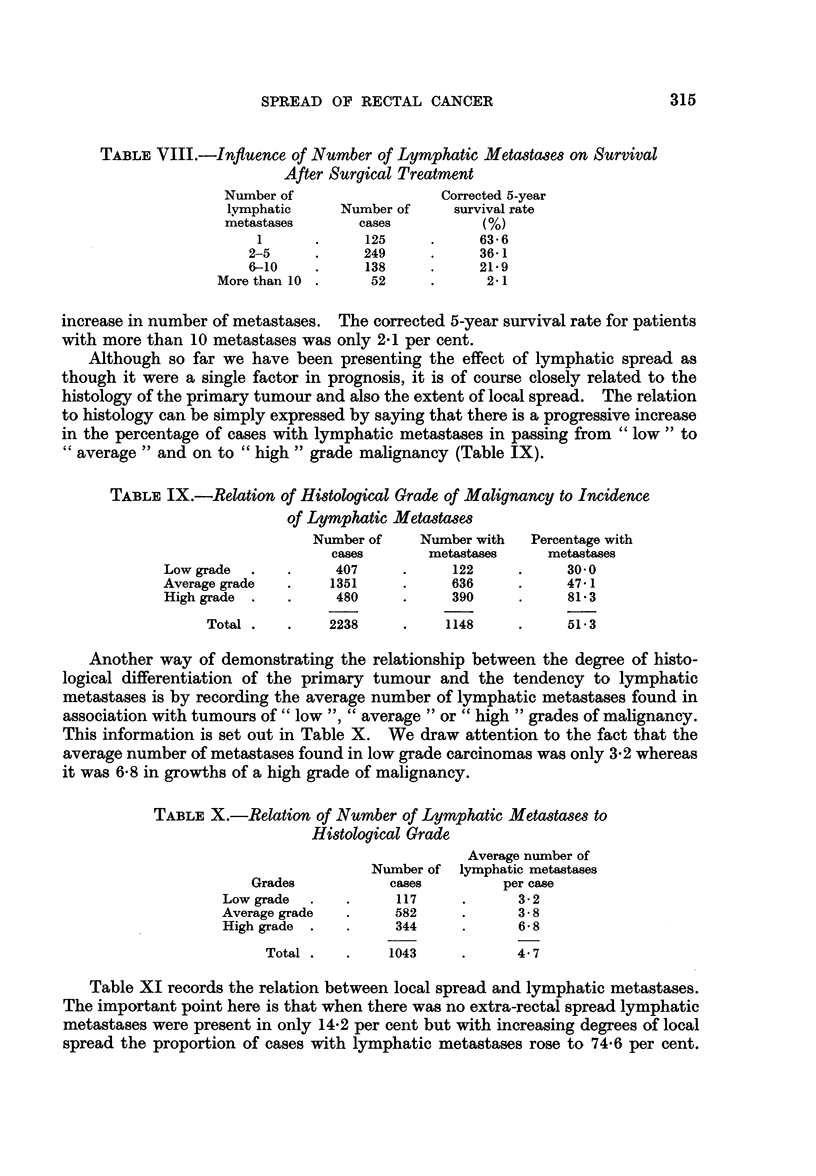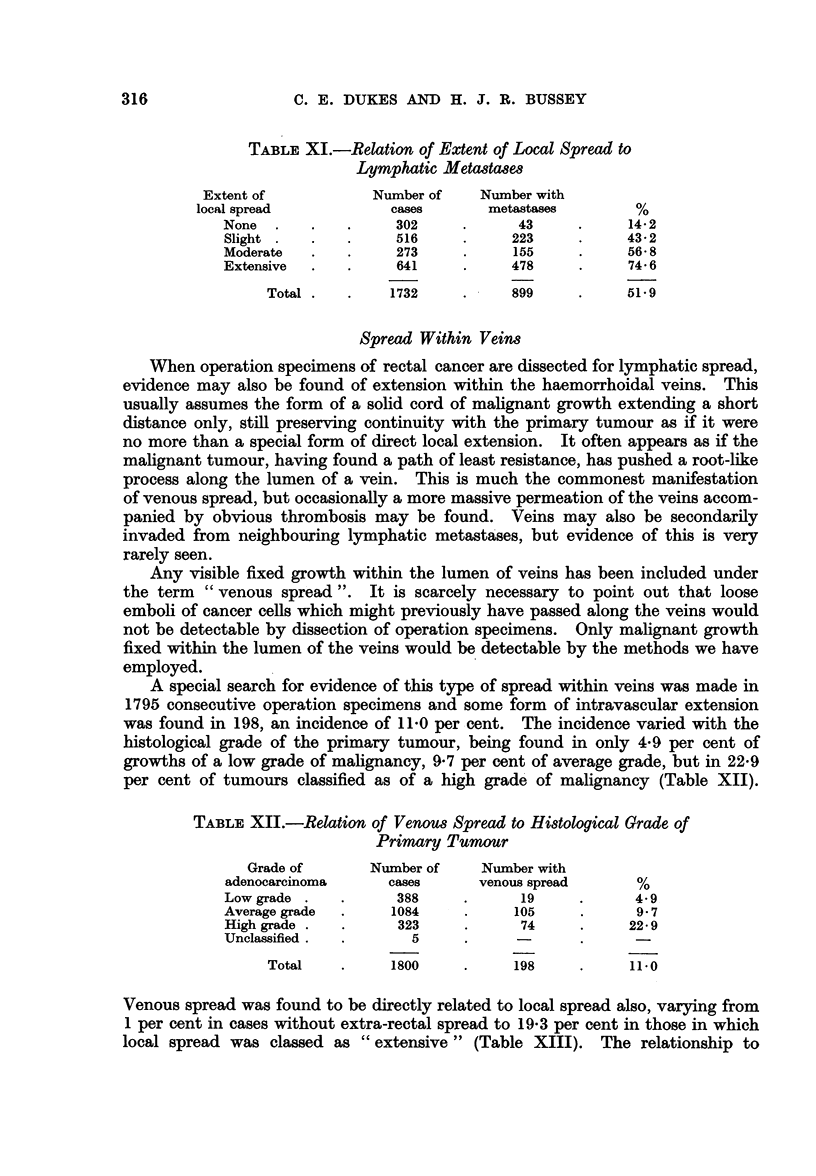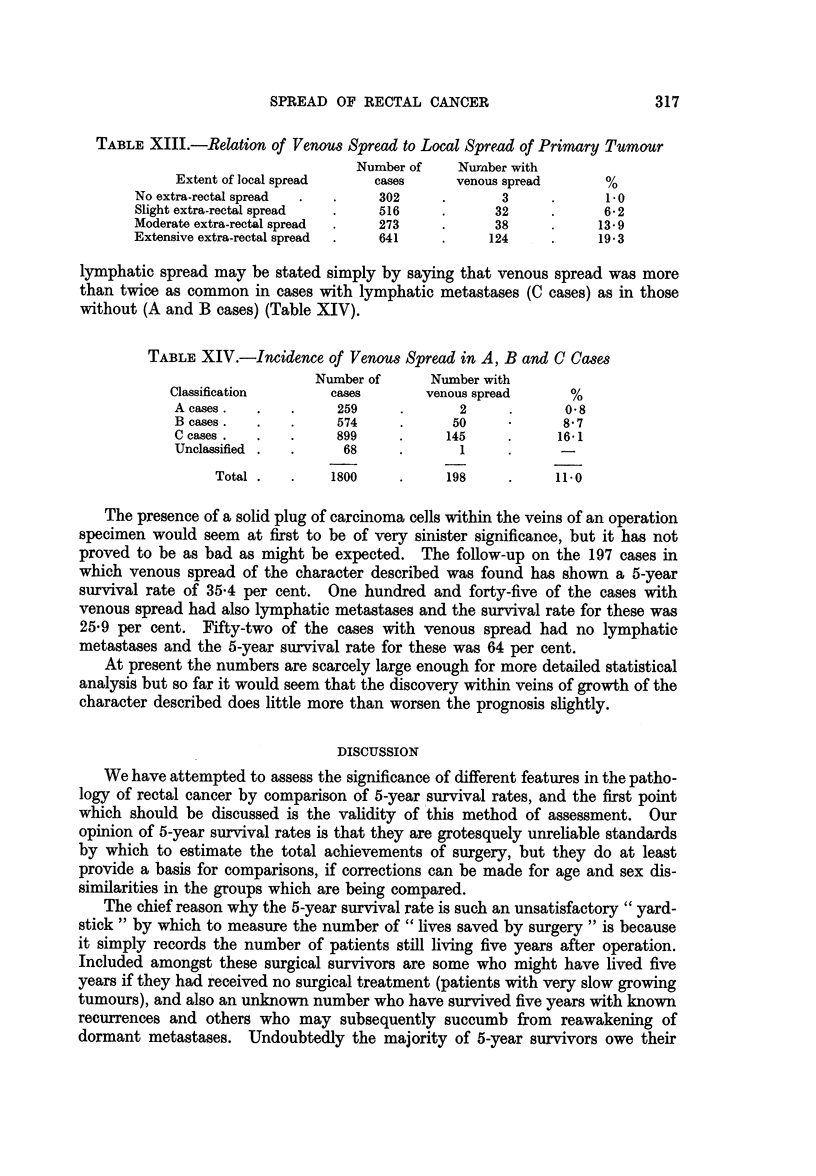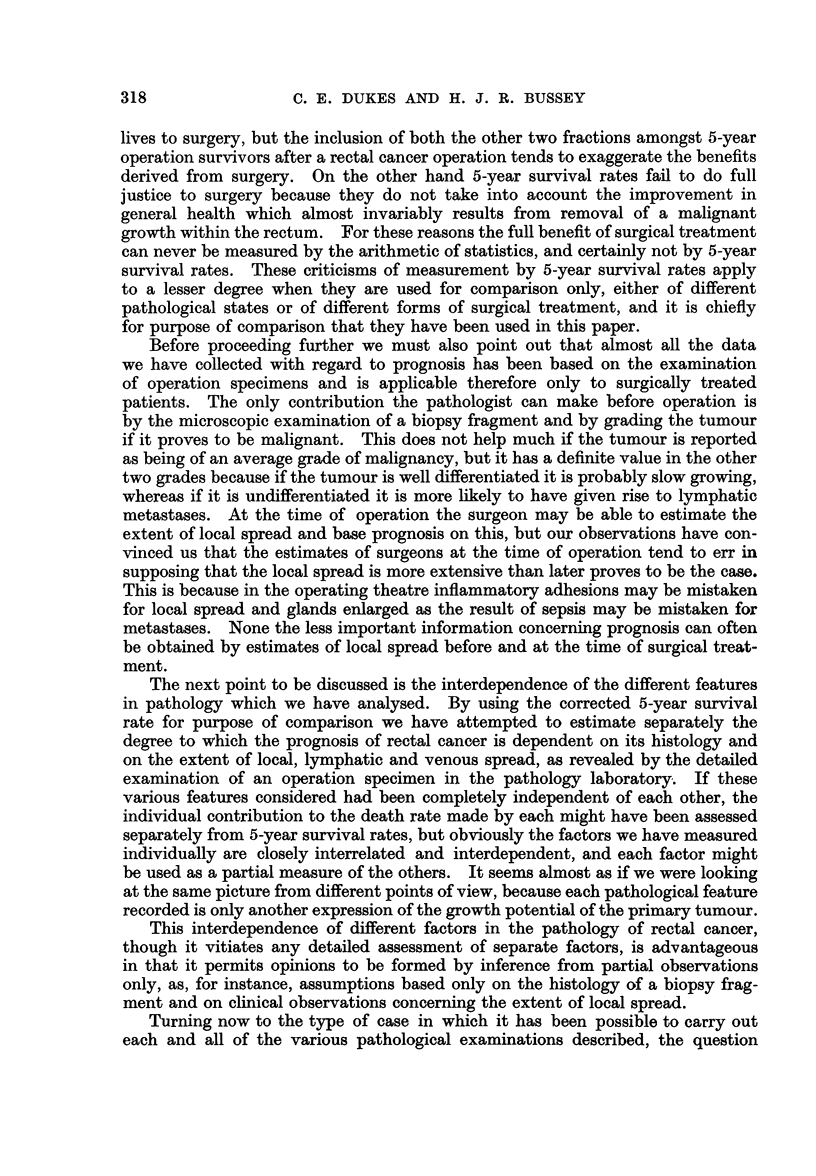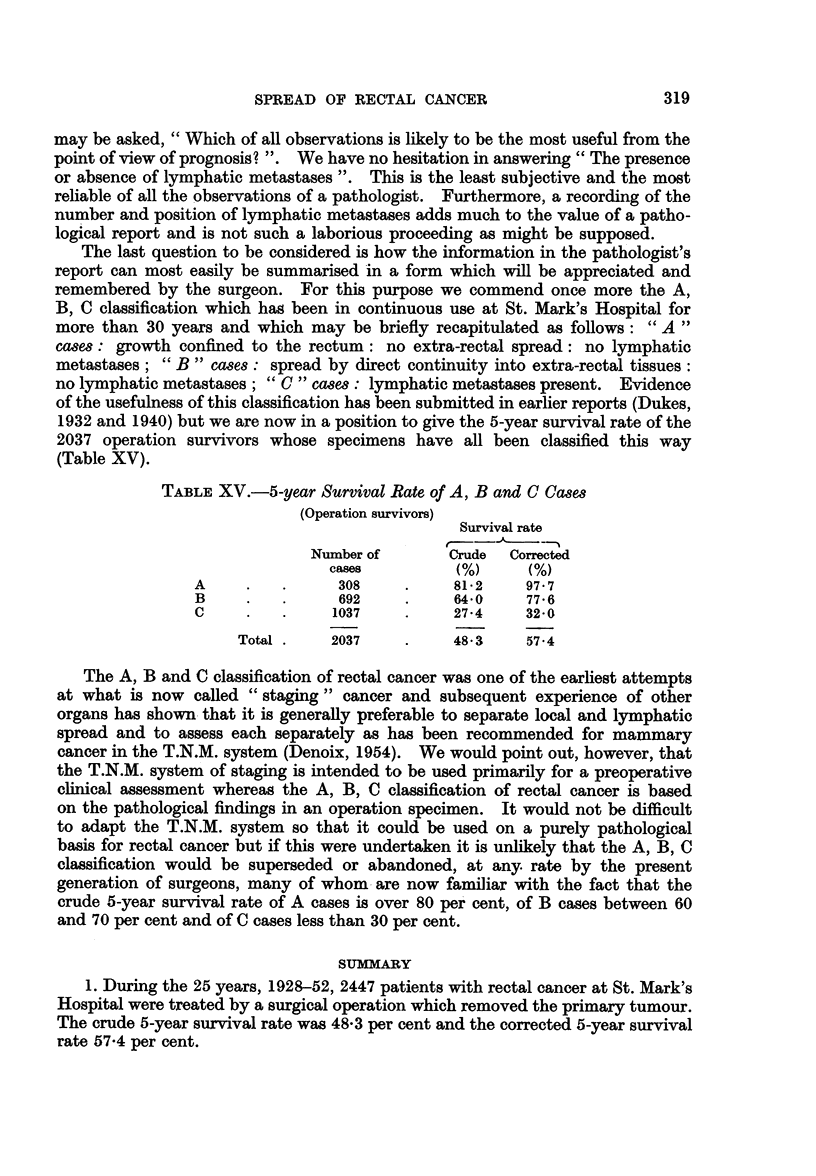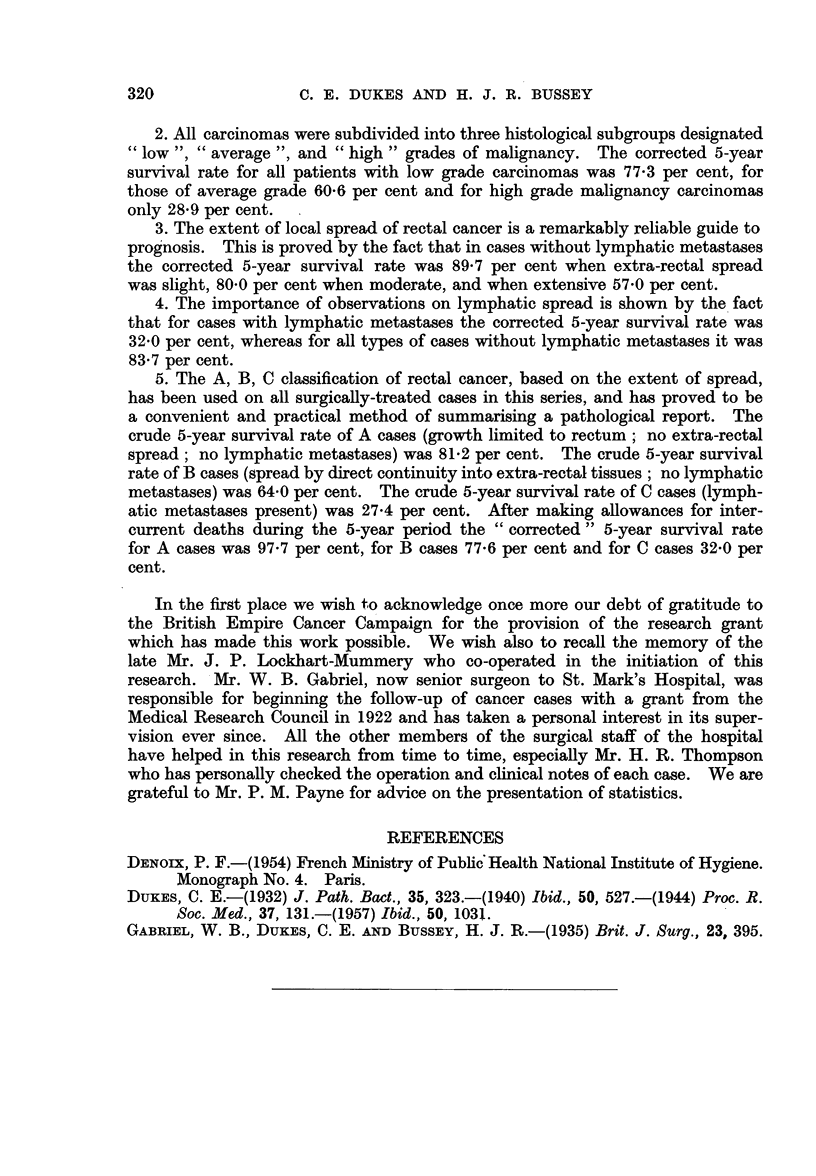# The Spread of Rectal Cancer and its Effect on Prognosis

**DOI:** 10.1038/bjc.1958.37

**Published:** 1958-09

**Authors:** C. E. Dukes, H. J. R. Bussey

## Abstract

**Images:**


### THE SPREAD OF RECTAL CANCER AND ITS EFFECT ON PROGNOSIS

C. E. DUKES AND H. J. R. BUSSEY

*From St. Mark's Hospital, City Road, London, E.C.1*

Received for publication July 30, 1958

It is well known that in all forms of cancer treated by radical surgery the patient's prognosis depends very much on the extent of local and lymphatic spread and on the likelihood of venous dissemination. To proceed further than this and to attempt to define the relative importance of each of these three methods of spread is only possible when a large series of carefully documented cases has been kept under observation for several years by a really efficient follow-up. This information is now available with regard to cases of rectal cancer treated by radical surgery at St. Mark's Hospital and the purpose of this paper is to record the degree to which the prognosis is influenced by the extent of local, lymphatic and venous spread.

St. Mark's Hospital is a special hospital for diseases of the colon and rectum, and since 1924 a research into the pathology and treatment of rectal cancer has been continued in the pathology department with the help of a grant from the British Empire Cancer Campaign. Some preliminary reports have already been published but the present analysis is the first comprehensive summary of this long-term research project and covers the 25 years 1928–52.

During these 25 years a total of 3596 rectal cancer patients were admitted to hospital or seen in the Out-patient Department. Of these, 2447 were treated by a surgical operation which removed the primary tumour. Every possible endeavour has been made to keep in touch with operation survivors, and only 28 have not been traced. These have been assumed to be dead, but since they constitute only 1·1 per cent of all cases they exert no appreciable effect on the statistics.

The crude 5-year survival rate of all patients treated by surgical removal of the primary tumour was 48·3 per cent. It might be assumed that this figure would provide a basic standard by which to measure the effect of various forms of spread subsequently to be considered, but crude survival rates are not altogether satisfactory for such a purpose because of variation in the age and sex composition of the groups of patients to be compared. When comparing groups of patients of dissimilar age and sex, it is more accurate to use " age and sex corrected " 5-year survival rates. This point will be appreciated when we recall that the crude 5-year survival rate simply records the percentage of individuals still living after 5 years and makes no distinction between deaths which have been due to cancer and deaths due to intercurrent diseases, the incidence of which is a function of both age and sex.

There is more than one statistical device for producing a corrected survival rate and the one adopted here was recommended by Dr. Percy Stocks and has already been described (Dukes, 1957). The effect of this correction on our figures has been generally to increase the crude survival rate by about one-fifth of its original value. Thus as already stated, the crude 5-year survival rate of all patients treated by surgical removal of the primary tumour was 48·3 per cent. The corrected 5-year survival rate was found to be 57·4 per cent and it is this figure which will be used for all the comparisons subsequently to be reported.

It should be noted that all survival rates presented in this paper are based on the total operation survivors within the specific group analysed, no distinction being made between those cases in which the growth was incompletely removed (palliative operations) and those where a cure could be hoped for (radical operations).

Further, it may be stated at this point that any apparent inconsistency in the total numbers in the tables is due to the exclusion, where necessary, of cases dying from the operation, of those with multiple intestinal malignancies, and of those where there was insufficient information for classification. Multiple intestinal cancer cases, which constitute about 5 per cent of the total, present special difficulties in regard to recording and interpretation and for this reason are being investigated as a separate group by one of us (H. J. R. B.).

### *Methods Used for Examination of Operation Specimens*

The examination of an operation specimen of rectal cancer provides the pathologist with an opportunity of making observations on the histology of the tumour ; the extent of local spread by direct continuity ; the presence, position and number of lymphatic metastases, and the evidence of spread within the lumen of veins. The prognostic significance of these will be considered in turn, but we must first point out that subsequent pathological investigations are made much easier if care is taken in the initial treatment of the operation specimen.

The plan adopted for the examination of operation specimens of rectal cancer and the dissection of the lymph nodes and blood vessels has already been described (Dukes, 1932 ; Gabriel, Dukes and Bussey, 1935 ; and Dukes, 1944), and may be briefly recapitulated as follows :

Immediately after removal, the specimen is sent to the Pathological Laboratory, where it is cut open along its anterior (anti-mesenteric) border, sewn on to a perforated metal frame and fixed in 10 per cent formalin solution for at least 48 hours. The fixed specimen is then photographed and dissected. The dissection begins at the point of division of the vascular pedicle and is directed at exposing the superior haemorrhoidal vessels and their branches until they disappear through the muscle coat into the submucosa. During this process most of the accompanying lymph nodes are discovered, the remainder being usually palpable in the thin wedges of fatty tissue left between the vessels. At the same time any growth extending along the veins is detected.

A natural-sized tracing of the specimen and its salient anatomical features is easily made by means of carbon paper placed beneath oiled silk. The lymph nodes are plotted on to this map in a numbered sequence and then taken for section, which is facilitated by pinning 15 or 20 nodes to a wooden block, the pins being removed after the block has been embedded in wax. After microscopical confirmation, the position of lymphatic metastases and any venous invasion are noted on the map, as is also the presence of local spread which is determined by a transverse cut through the centre of the tumour.

### *Histological Grading*

In this paper we do not intend to discuss details of histological classification but only to say that it has been our practice to subdivide rectal carcinomas into three histological subgroups, designated as "low", "average" and "high" grades of malignancy—these distinctions being based on cellular arrangement and differentiation (Fig. 1, 2 and 3). There are, of course, no sharply defined boundaries to the grades and grading is an artificial division into three arbitrary groups. For this reason some difficulty may be expected in the placing of tumours which appear to be intermediate in character but in spite of these objections grading has proved to be a useful method of subdividing rectal cancers and of considerable value in relation to prognosis.

The dependence of prognosis on histology is shown by the fact that the corrected 5-year survival rate of all patients reported as having low grade carcinomas was 77·3 per cent, for those of average grade 60·6 per cent and for high grade malignancy tumours 28·9 per cent (Table I).

TABLE I.—*Relation of Grade of Malignancy to 5-year Survival After Surgical Treatment*

All cases

| | Number of cases | Corrected 5-year survival rate (%) |
|---|---|---|
| Low grade | 407 | 77·3 |
| Average grade | 1266 | 60·6 |
| High grade | 424 | 28·9 |

In passing it may be noted that there are significant differences in the average age of groups of patients whose tumours were classed as low, average and high grade of malignancy, the average age of men with low grade malignancy tumours being 4·9 years higher than for those with high malignancy growths (Table II).

TABLE II.—*Average Age in Years of Patients Graded on Basis of Histology*

| | Males | Females |
|---|---|---|
| Low grade malignancy | $61\cdot9\pm0\cdot6$ | $61\cdot3\pm0\cdot9$ |
| Average grade malignancy | $60\cdot5\pm0\cdot3$ | $58\cdot3\pm0\cdot5$ |
| High grade malignancy | $57\cdot0\pm0\cdot7$ | $53\cdot8\pm1\cdot1$ |

Difference between low and average (males) $= 1\cdot4\pm0\cdot7$
" " average and high (males) $= 3\cdot5\pm0\cdot7$
" " low and average (females) $= 3\cdot0\pm1\cdot1$
" " average and high (females) $= 4\cdot5\pm1\cdot1$

In women the differences were even greater, the average age of those patients with low grade malignancy tumours being as much as 7·5 years higher than the average for those with high malignancy.

### *Local Spread*

We use the term "local spread" to describe spread *by direct continuity* from the original point or points of origin of a malignant growth. Local spread may vary greatly in extent. It may be classified as (1) confined to the bowel wall

(no extra-rectal spread); (2) commencing to invade the extra-rectal tissues (slight local spread); (3) well established in the mesentery (moderate spread) or (4) deeply invasive, possibly into neighbouring organs (extensive spread).

TABLE III.—*Relationship of Grade of Malignancy to Extent of Local Spread*

| | No extra-rectal spread (%) | Slight spread (%) | Moderate spread (%) | Extensive spread (%) |
|---|---|---|---|---|
| Low grade | 37·1 | 21·1 | 18·7 | 14·5 |
| Average grade | 58·3 | 68·2 | 65·9 | 52·9 |
| High grade | 4·6 | 10·7 | 15·4 | 32·6 |

As might be expected the degree of local spread is closely related to the degree of histological differentiation of the primary tumour, a relationship demonstrated in Table III which records the percentage of low, average and high grades of malignancy associated with no extra-rectal spread and in those in which local spread was slight, moderate or extensive. For example, growths of a high grade of malignancy constituted only 4·6 per cent of tumours without extra-rectal spread, whereas high grade malignancies were found to the extent of 32·6 per cent in tumours with extensive extra-rectal spread.

TABLE IV.—*Relationship Between Local Spread and 5-year Survival Rates After Surgical Treatment*

Cases without lymphatic metastases

| | Number of cases | Corrected 5-year survival rate (%) |
|---|---|---|
| Slight extra-rectal spread | 266 | 89·7 |
| Moderate extra-rectal spread | 109 | 80·0 |
| Extensive extra-rectal spread | 148 | 57·0 |

To assess the prognostic significance of local spread we have confined the analysis to cases in which no lymphatic metastases were found when the operation specimen was dissected, and in Table IV we give the survival rates for three subdivisions of local spread in a consecutive series of 523 operation survivors without lymphatic metastases. Increasing local spread also increases the liability to dissemination of cancer cells by lymphatic and venous channels and evidence for this will be provided later. It may be mentioned here that the 5-year survival rate falls to less than 10 per cent when the local spread involves the peritoneum. This is not unexpected as this form of spread is found to occur most commonly in cases of high grade malignancy with extensive lymphatic metastases.

The effect on prognosis of histology and of extent of spread has been considered separately in Tables I and IV. Their effect in combination is set out in Table V from which it may be inferred that in each subdivision of histological

---

EXPLANATION OF PLATE

FIG. 1.—Rectal carcinoma. Low grade malignancy. × 140.
FIG. 2.—Rectal carcinoma. Average grade malignancy. × 140.
FIG. 3.—Rectal carcinoma. High grade malignancy. × 140.

1

2

3

Dukes and Bussey.

TABLE V.—*Relation of Grade of Malignancy and Extent of Spread to 5-year Survival After Surgical Treatment*

| Grade | *Nil* spread | | Slight spread | | Moderate spread | | Extensive spread | |
|---|---|---|---|---|---|---|---|---|
| | Number of cases | 5-year survival (%) | Number of cases | 5-year survival (%) | Number of cases | 5-year survival (%) | Number of cases | 5-year survival (%) |
| Low | 102 | 100·0 | 101 | 83·5 | 48 | 57·2 | 89 | 48·0 |
| Average | 159 | 97·6 | 321 | 77·7 | 176 | 60·2 | 301 | 28·4 |
| High | 13 | 62·3 | 50 | 43·5 | 38 | 42·4 | 181 | 16·0 |

grade the survival rate decreases with increased local spread. Moreover, apart from a minor exception in the moderate group, each degree of local spread shows a lessening of survival with increasing malignancy of the tumour. That this close connection should exist is not surprising when it is remembered that histological grading is little more than an attempt to assess microscopically the invasive character of a tumour.

Before passing on to consider lymphatic and venous spread we draw attention to the fact that the estimation of the extent of local spread is a relatively simple procedure and quickly provides valuable information with respect to prognosis.

### *Lymphatic Spread*

In investigating the general effect on prognosis of lymphatic metastases we have calculated the survival rates of men and women separately because there are slight differences in the incidence of lymphatic spread in the two sexes. The corrected 5-year survival rate for men without lymphatic metastases (A and B cases) was 82·5 per cent and this was reduced to 31·1 per cent for those with lymphatic metastases (C cases). The corresponding figures for women were: for those without metastases 86·2 per cent and for those with metastases 33·1 per cent (Table VI).

TABLE VI.—*5-year Survival Rates of Cases With and Without Lymphatic Metastases*

| | Number of cases | Corrected 5-year survival rate (%) |
|---|---|---|
| *Men*— | | |
| Without metastases (A and B cases) | 683 | 82·5 |
| With metastases (C cases) | 641 | 31·1 |
| *Women*— | | |
| Without metastases (A and B cases) | 317 | 86·2 |
| With metastases (C cases) | 396 | 33·1 |
| *Both sexes*— | | |
| Without metastases (A and B cases) | 1000 | 83·7 |
| With metastases (C cases) | 1037 | 32·0 |

The dividing of all cases into two groups in this way according to whether or not lymphatic metastases have been found tends to exaggerate the prognostic significance of lymphatic spread and to disguise the fact that if lymphatic spread is still at an early stage the prognosis may still be relatively good. This becomes clear when cases with metastases are further subdivided on the basis of the position and number of the metastases.

To illustrate the importance of the position of metastases, all " C " cases were further subdivided into two groups. If only the regional lymph nodes contained metastases the case was classified as " C. 1 ", whereas if there was more extensive lymphatic spread involving the nodes at the point of ligature of the blood vessels the case was described as " C. 2 " (Fig. 4). A comparison of the survival rates

FIG. 4.—Subdivision of cases with lymphatic metastases into C. 1 and C. 2.

TABLE VII.—*Comparison of Survival Rates of C.1 and C.2 Cases*

| | Number of cases | Corrected 5-year survival rate (%) |
|---|---|---|
| C. 1 cases | 680 | 40·9 |
| C. 2 cases | 282 | 13·6 |
| Unclassified | 75 | 19·6 |
| Total | 1037 | 32·0 |

of these two groups is recorded in Table VII, from which it will be seen that the corrected 5-year survival rate for C. 1 patients was 40·9 per cent but only 13·6 per cent for the C. 2 patients. Also it may be pointed out that the number of C. 1 cases was more than twice as great as the C. 2, which in itself suggests that upward spread along the haemorrhoidal chain of lymph nodes is normally a slow process.

To investigate the significance of the number of metastases, cases with lymphatic spread have been further subdivided into convenient groups as follows—one metastasis only, 2–5 metastases, 6–9 metastases, and 10 or more metastases. The analysis of these subgroups is recorded in Table VIII from which it will be seen that the corrected 5-year survival rate of those patients in whom only one metastasis was found was 63·6 per cent but after this prognosis worsened with

TABLE VIII.—*Influence of Number of Lymphatic Metastases on Survival After Surgical Treatment*

| Number of lymphatic metastases | Number of cases | Corrected 5-year survival rate (%) |
|---|---|---|
| 1 | 125 | 63·6 |
| 2–5 | 249 | 36·1 |
| 6–10 | 138 | 21·9 |
| More than 10 | 52 | 2·1 |

increase in number of metastases. The corrected 5-year survival rate for patients with more than 10 metastases was only 2·1 per cent.

Although so far we have been presenting the effect of lymphatic spread as though it were a single factor in prognosis, it is of course closely related to the histology of the primary tumour and also the extent of local spread. The relation to histology can be simply expressed by saying that there is a progressive increase in the percentage of cases with lymphatic metastases in passing from " low " to " average " and on to " high " grade malignancy (Table IX).

TABLE IX.—*Relation of Histological Grade of Malignancy to Incidence of Lymphatic Metastases*

| | Number of cases | Number with metastases | Percentage with metastases |
|---|---|---|---|
| Low grade | 407 | 122 | 30·0 |
| Average grade | 1351 | 636 | 47·1 |
| High grade | 480 | 390 | 81·3 |
| Total | 2238 | 1148 | 51·3 |

Another way of demonstrating the relationship between the degree of histological differentiation of the primary tumour and the tendency to lymphatic metastases is by recording the average number of lymphatic metastases found in association with tumours of " low ", " average " or " high " grades of malignancy. This information is set out in Table X. We draw attention to the fact that the average number of metastases found in low grade carcinomas was only 3·2 whereas it was 6·8 in growths of a high grade of malignancy.

TABLE X.—*Relation of Number of Lymphatic Metastases to Histological Grade*

| Grades | Number of cases | Average number of lymphatic metastases per case |
|---|---|---|
| Low grade | 117 | 3·2 |
| Average grade | 582 | 3·8 |
| High grade | 344 | 6·8 |
| Total | 1043 | 4·7 |

Table XI records the relation between local spread and lymphatic metastases. The important point here is that when there was no extra-rectal spread lymphatic metastases were present in only 14·2 per cent but with increasing degrees of local spread the proportion of cases with lymphatic metastases rose to 74·6 per cent.

TABLE XI.—*Relation of Extent of Local Spread to Lymphatic Metastases*

| Extent of local spread | Number of cases | Number with metastases | % |
|---|---|---|---|
| None | 302 | 43 | 14·2 |
| Slight | 516 | 223 | 43·2 |
| Moderate | 273 | 155 | 56·8 |
| Extensive | 641 | 478 | 74·6 |
| Total | 1732 | 899 | 51·9 |

### *Spread Within Veins*

When operation specimens of rectal cancer are dissected for lymphatic spread, evidence may also be found of extension within the haemorrhoidal veins. This usually assumes the form of a solid cord of malignant growth extending a short distance only, still preserving continuity with the primary tumour as if it were no more than a special form of direct local extension. It often appears as if the malignant tumour, having found a path of least resistance, has pushed a root-like process along the lumen of a vein. This is much the commonest manifestation of venous spread, but occasionally a more massive permeation of the veins accompanied by obvious thrombosis may be found. Veins may also be secondarily invaded from neighbouring lymphatic metastases, but evidence of this is very rarely seen.

Any visible fixed growth within the lumen of veins has been included under the term " venous spread ". It is scarcely necessary to point out that loose emboli of cancer cells which might previously have passed along the veins would not be detectable by dissection of operation specimens. Only malignant growth fixed within the lumen of the veins would be detectable by the methods we have employed.

A special search for evidence of this type of spread within veins was made in 1795 consecutive operation specimens and some form of intravascular extension was found in 198, an incidence of 11·0 per cent. The incidence varied with the histological grade of the primary tumour, being found in only 4·9 per cent of growths of a low grade of malignancy, 9·7 per cent of average grade, but in 22·9 per cent of tumours classified as of a high grade of malignancy (Table XII).

TABLE XII.—*Relation of Venous Spread to Histological Grade of Primary Tumour*

| Grade of adenocarcinoma | Number of cases | Number with venous spread | % |
|---|---|---|---|
| Low grade | 388 | 19 | 4·9 |
| Average grade | 1084 | 105 | 9·7 |
| High grade | 323 | 74 | 22·9 |
| Unclassified | 5 | — | — |
| Total | 1800 | 198 | 11·0 |

Venous spread was found to be directly related to local spread also, varying from 1 per cent in cases without extra-rectal spread to 19·3 per cent in those in which local spread was classed as " extensive " (Table XIII). The relationship to

TABLE XIII.—*Relation of Venous Spread to Local Spread of Primary Tumour*

| Extent of local spread | Number of cases | Number with venous spread | % |
|---|---|---|---|
| No extra-rectal spread | 302 | 3 | 1·0 |
| Slight extra-rectal spread | 516 | 32 | 6·2 |
| Moderate extra-rectal spread | 273 | 38 | 13·9 |
| Extensive extra-rectal spread | 641 | 124 | 19·3 |

lymphatic spread may be stated simply by saying that venous spread was more than twice as common in cases with lymphatic metastases (C cases) as in those without (A and B cases) (Table XIV).

TABLE XIV.—*Incidence of Venous Spread in A, B and C Cases*

| Classification | Number of cases | Number with venous spread | % |
|---|---|---|---|
| A cases | 259 | 2 | 0·8 |
| B cases | 574 | 50 | 8·7 |
| C cases | 899 | 145 | 16·1 |
| Unclassified | 68 | 1 | — |
| Total | 1800 | 198 | 11·0 |

The presence of a solid plug of carcinoma cells within the veins of an operation specimen would seem at first to be of very sinister significance, but it has not proved to be as bad as might be expected. The follow-up on the 197 cases in which venous spread of the character described was found has shown a 5-year survival rate of 35·4 per cent. One hundred and forty-five of the cases with venous spread had also lymphatic metastases and the survival rate for these was 25·9 per cent. Fifty-two of the cases with venous spread had no lymphatic metastases and the 5-year survival rate for these was 64 per cent.

At present the numbers are scarcely large enough for more detailed statistical analysis but so far it would seem that the discovery within veins of growth of the character described does little more than worsen the prognosis slightly.

### DISCUSSION

We have attempted to assess the significance of different features in the pathology of rectal cancer by comparison of 5-year survival rates, and the first point which should be discussed is the validity of this method of assessment. Our opinion of 5-year survival rates is that they are grotesquely unreliable standards by which to estimate the total achievements of surgery, but they do at least provide a basis for comparisons, if corrections can be made for age and sex dissimilarities in the groups which are being compared.

The chief reason why the 5-year survival rate is such an unsatisfactory "yardstick" by which to measure the number of "lives saved by surgery" is because it simply records the number of patients still living five years after operation. Included amongst these surgical survivors are some who might have lived five years if they had received no surgical treatment (patients with very slow growing tumours), and also an unknown number who have survived five years with known recurrences and others who may subsequently succumb from reawakening of dormant metastases. Undoubtedly the majority of 5-year survivors owe their lives to surgery, but the inclusion of both the other two fractions amongst 5-year operation survivors after a rectal cancer operation tends to exaggerate the benefits derived from surgery. On the other hand 5-year survival rates fail to do full justice to surgery because they do not take into account the improvement in general health which almost invariably results from removal of a malignant growth within the rectum. For these reasons the full benefit of surgical treatment can never be measured by the arithmetic of statistics, and certainly not by 5-year survival rates. These criticisms of measurement by 5-year survival rates apply to a lesser degree when they are used for comparison only, either of different pathological states or of different forms of surgical treatment, and it is chiefly for purpose of comparison that they have been used in this paper.

Before proceeding further we must also point out that almost all the data we have collected with regard to prognosis has been based on the examination of operation specimens and is applicable therefore only to surgically treated patients. The only contribution the pathologist can make before operation is by the microscopic examination of a biopsy fragment and by grading the tumour if it proves to be malignant. This does not help much if the tumour is reported as being of an average grade of malignancy, but it has a definite value in the other two grades because if the tumour is well differentiated it is probably slow growing, whereas if it is undifferentiated it is more likely to have given rise to lymphatic metastases. At the time of operation the surgeon may be able to estimate the extent of local spread and base prognosis on this, but our observations have convinced us that the estimates of surgeons at the time of operation tend to err in supposing that the local spread is more extensive than later proves to be the case. This is because in the operating theatre inflammatory adhesions may be mistaken for local spread and glands enlarged as the result of sepsis may be mistaken for metastases. None the less important information concerning prognosis can often be obtained by estimates of local spread before and at the time of surgical treatment.

The next point to be discussed is the interdependence of the different features in pathology which we have analysed. By using the corrected 5-year survival rate for purpose of comparison we have attempted to estimate separately the degree to which the prognosis of rectal cancer is dependent on its histology and on the extent of local, lymphatic and venous spread, as revealed by the detailed examination of an operation specimen in the pathology laboratory. If these various features considered had been completely independent of each other, the individual contribution to the death rate made by each might have been assessed separately from 5-year survival rates, but obviously the factors we have measured individually are closely interrelated and interdependent, and each factor might be used as a partial measure of the others. It seems almost as if we were looking at the same picture from different points of view, because each pathological feature recorded is only another expression of the growth potential of the primary tumour.

This interdependence of different factors in the pathology of rectal cancer, though it vitiates any detailed assessment of separate factors, is advantageous in that it permits opinions to be formed by inference from partial observations only, as, for instance, assumptions based only on the histology of a biopsy fragment and on clinical observations concerning the extent of local spread.

Turning now to the type of case in which it has been possible to carry out each and all of the various pathological examinations described, the question may be asked, " Which of all observations is likely to be the most useful from the point of view of prognosis? ". We have no hesitation in answering " The presence or absence of lymphatic metastases ". This is the least subjective and the most reliable of all the observations of a pathologist. Furthermore, a recording of the number and position of lymphatic metastases adds much to the value of a pathological report and is not such a laborious proceeding as might be supposed.

The last question to be considered is how the information in the pathologist's report can most easily be summarised in a form which will be appreciated and remembered by the surgeon. For this purpose we commend once more the A, B, C classification which has been in continuous use at St. Mark's Hospital for more than 30 years and which may be briefly recapitulated as follows: " *A* " *cases:* growth confined to the rectum: no extra-rectal spread: no lymphatic metastases; " *B* " *cases:* spread by direct continuity into extra-rectal tissues: no lymphatic metastases; " *C* " *cases:* lymphatic metastases present. Evidence of the usefulness of this classification has been submitted in earlier reports (Dukes, 1932 and 1940) but we are now in a position to give the 5-year survival rate of the 2037 operation survivors whose specimens have all been classified this way (Table XV).

TABLE XV.—*5-year Survival Rate of A, B and C Cases*

(Operation survivors)

| | Number of cases | Survival rate: Crude (%) | Survival rate: Corrected (%) |
|---|---|---|---|
| A | 308 | 81·2 | 97·7 |
| B | 692 | 64·0 | 77·6 |
| C | 1037 | 27·4 | 32·0 |
| Total | 2037 | 48·3 | 57·4 |

The A, B and C classification of rectal cancer was one of the earliest attempts at what is now called " staging " cancer and subsequent experience of other organs has shown that it is generally preferable to separate local and lymphatic spread and to assess each separately as has been recommended for mammary cancer in the T.N.M. system (Denoix, 1954). We would point out, however, that the T.N.M. system of staging is intended to be used primarily for a preoperative clinical assessment whereas the A, B, C classification of rectal cancer is based on the pathological findings in an operation specimen. It would not be difficult to adapt the T.N.M. system so that it could be used on a purely pathological basis for rectal cancer but if this were undertaken it is unlikely that the A, B, C classification would be superseded or abandoned, at any rate by the present generation of surgeons, many of whom are now familiar with the fact that the crude 5-year survival rate of A cases is over 80 per cent, of B cases between 60 and 70 per cent and of C cases less than 30 per cent.

### SUMMARY

1. During the 25 years, 1928–52, 2447 patients with rectal cancer at St. Mark's Hospital were treated by a surgical operation which removed the primary tumour. The crude 5-year survival rate was 48·3 per cent and the corrected 5-year survival rate 57·4 per cent.

2. All carcinomas were subdivided into three histological subgroups designated "low", "average", and "high" grades of malignancy. The corrected 5-year survival rate for all patients with low grade carcinomas was 77·3 per cent, for those of average grade 60·6 per cent and for high grade malignancy carcinomas only 28·9 per cent.

3. The extent of local spread of rectal cancer is a remarkably reliable guide to prognosis. This is proved by the fact that in cases without lymphatic metastases the corrected 5-year survival rate was 89·7 per cent when extra-rectal spread was slight, 80·0 per cent when moderate, and when extensive 57·0 per cent.

4. The importance of observations on lymphatic spread is shown by the fact that for cases with lymphatic metastases the corrected 5-year survival rate was 32·0 per cent, whereas for all types of cases without lymphatic metastases it was 83·7 per cent.

5. The A, B, C classification of rectal cancer, based on the extent of spread, has been used on all surgically-treated cases in this series, and has proved to be a convenient and practical method of summarising a pathological report. The crude 5-year survival rate of A cases (growth limited to rectum ; no extra-rectal spread ; no lymphatic metastases) was 81·2 per cent. The crude 5-year survival rate of B cases (spread by direct continuity into extra-rectal tissues ; no lymphatic metastases) was 64·0 per cent. The crude 5-year survival rate of C cases (lymphatic metastases present) was 27·4 per cent. After making allowances for intercurrent deaths during the 5-year period the "corrected" 5-year survival rate for A cases was 97·7 per cent, for B cases 77·6 per cent and for C cases 32·0 per cent.

In the first place we wish to acknowledge once more our debt of gratitude to the British Empire Cancer Campaign for the provision of the research grant which has made this work possible. We wish also to recall the memory of the late Mr. J. P. Lockhart-Mummery who co-operated in the initiation of this research. Mr. W. B. Gabriel, now senior surgeon to St. Mark's Hospital, was responsible for beginning the follow-up of cancer cases with a grant from the Medical Research Council in 1922 and has taken a personal interest in its supervision ever since. All the other members of the surgical staff of the hospital have helped in this research from time to time, especially Mr. H. R. Thompson who has personally checked the operation and clinical notes of each case. We are grateful to Mr. P. M. Payne for advice on the presentation of statistics.

### REFERENCES

Denoix, P. F.—(1954) French Ministry of Public Health National Institute of Hygiene. Monograph No. 4. Paris.

Dukes, C. E.—(1932) *J. Path. Bact.*, **35**, 323.—(1940) *Ibid.*, **50**, 527.—(1944) *Proc. R. Soc. Med.*, **37**, 131.—(1957) *Ibid.*, **50**, 1031.

Gabriel, W. B., Dukes, C. E. and Bussey, H. J. R.—(1935) *Brit. J. Surg.*, **23**, 395.